# The Book World of Medicine and Science

**Published:** 1907-09-28

**Authors:** 


### The Book World of Medicine and Science.

A System of Medicine. By Eminent Authorities in Great Britain, the United States, and the Continent. Edited by Wm. Osler, M.D., F.R.S., assisted by Thomas McCrae, M.D., F.R.C.P. Volume II. (Henry Frowde, Oxford University Press; Hodder and Stoughton, London, 1907.)

In our issue of August 10 we had the pleasure of noticing the first volume of this new system of medicine and of expressing our appreciation both of its scientific tone and of its adaptation to the needs of the practitioner. The volume now before us is concerned with the infectious diseases, and there is no difficulty in tracing in it an imprint of the same features which were so readily recognised in its predecessor. The various contributors have evidently approached, and carried through, their several tasks in harmony with a plan which may well be the expression of an editorial ideal, and the result is an eminently satisfactory one. It is, indeed, not too much to say that this is one of the most completely ordered and practical volumes on the subject of infectious diseases that can be consulted. Even if the reader for any reason cannot arrange to subscribe for the entire system, he will certainly be well advised to place this second volume on his shelves. The opportunities for systematic study afforded by the practice of isolating and treating cases of these diseases in public hospitals have led to advances both in theory and practice, and these advances in the present work secure ample recognition. At the same time, it is evident that great pains have been taken to obtain clearness and fullness in exposition, so that the various chapters are, almost without exception, as readable as they are valuable.

Chapter I. is entitled "An Introduction to the Study of Infectious Diseases," and is from the pen of Dr. Ludvig Hektoen. It is an admirable statement of the modern doctrines of infection and immunity, and includes a discussion of all the more recent views on these subjects, while at the same time it avoids the severity of an unduly technical analysis. Even those whose work is not obviously associated with the subjects of which Dr. Hektoen writes will read his contribution with pleasure. In Chapters II. to VI. Dr. McCrae deals with typhoid fever, and his contribution is one of the most interesting and important in the whole volume. In particular we would venture to commend the sections dealing with the prevention of the disease and with the diagnosis of perforation of the bowel. Personally, we should like to have had a little more information about the cases now spoken of as "renal typhoid." The true nature of these is very apt to be overlooked, and they carry danger both to the individual patient and to the general community. Dr. McCrae is also responsible for the chapters on typhus fever, to which, significantly enough, are allotted only fourteen pages, as compared with the 160 pages occupied by typhoid fever; he is able to report that there have been no outbreaks of this disease in the United States for over ten years. The same author contributes a short chapter on relapsing fever. Small-pox and vaccination are discussed very fully in chapters written respectively by Dr. Wm. T. Councilman and Professor Geo. Dock. Dr. Councilman also writes on chicken-pox, and each of his articles is illustrated by a number of very successful plates. The article on scarlet fever is from the pen of Dr. Jno. H. McCollom, and is an ably written and well illustrated contribution. Incidentally the author discusses the so-called "fourth disease," and expresses an opinion that the existence of this has not been proved. Both in this section and in that dealing with the treatment of typhoid fever great stress is placed upon the importance of encouraging the patient to drink large quantities of water. Dr. McCollom does not accept the opinion which has been advanced that infection is not spread by the desquamating epithelium, but solely by discharge from the mucous membranes. At the same time, he is disposed to restrict a capacity for infection to the primary desquamation. "There is very grave doubt," he writes, "if the secondary desquamation is infective." In dealing with the question of the transmission of the disease through the agency of milk, it is somewhat curious, in view of certain sensational statements prevalent in this country, to read: "In London the medical supervision of milk farms is very carefully conducted. In this country it would be well, for the health of the community, if the example of London were followed." Two points may be noted in the remarks relating to the treatment of nephritis as a complication of scarlet fever. The one is that "pilocarpine is of doubtful advantage," and the other the absence of any recommendation of the value of leeches applied to the loins at the outset of the attack; we fancy neither of these positions will meet with universal acceptance. In the chapters on "Measles" and "Rubella" Dr. Jno. Ruhräh maintains the high standard set in the earlier sections; his descriptions of the eruptions are very successful, and he deals with the various aspects of treatment in a thoroughly efficient manner. Here again an opinion is expressed that the existence of the "fourth disease" described by Dr. Clement Dukes has yet to be proved. Diphtheria is adequately dealt with by Dr. McCollom, though we confess we would fain have seen a more detailed attempt to establish a differential diagnosis between tonsillitis and diphtheria on clinical grounds. Dr. McCollom emphasises the value of the bacteriological method when this is rightly performed, and he doubts, in opposition to other statements, whether the specific bacillus is ever found in the throat of healthy persons. In accordance with modern views lobar pneumonia and acute rheumatism (the latter by Dr. Poynton) are here included among the infectious diseases. A very full and valuable account of the former is contributed by Dr. Jno. H. Musser and George W. Norris. On the question of blood-letting in pneumonia Dr. Musser says he "has never resorted to it," though there are conditions in which he thinks it might produce good results. On the other hand, he emphasises the value of dry-cupping. "The cups," he says, "should be applied all over both front and back to the number of twenty or thirty; this should be done early, and repeated every six or eight hours as long as pain persists, the dyspnœa or oppression continues, or the respiration rate rises. After cupping, the pain may further be relieved by *thorough strapping of the chest*." So far as we know, this procedure is not adopted on this side of the Atlantic. Lack of space forbids us even to mention the other valuable articles which this volume contains. They well deserve recognition, and we have no doubt they will receive it from all those who consult them. Altogether, the second volume of Professor Osler's "System" fully realises the expectations formed with regard to it. The work, without question, is going to prove a highly useful and authoritative record covering the whole field of medicine.

---

Hay Fever, Hay Asthma: Its Causes, Diagnosis, and Treatment. By William Lloyd, F.R.C.S. (London: Henry J. Glaisher. 1907. Price 3s. 6d. net.)

The practical direction of Mr. Lloyd's essay is to emphasise the relations between hay fever and "the presence of hypersensitive areas in the nasal mucous membrane." On this is based a claim for local treatment of the unduly sensitive parts, and the author quotes a number of cases which go to support this claim. The essay is well written, and includes an interesting sketch of the various medical opinions which have been expressed on the nature of hay fever.

